# Supplementary material for: Strain-regulated Gibbs free energy enables reversible redox chemistry of chalcogenides for sodium ion batteries
Source: Nat Commun. 2022 Sep 23;13:5588. doi: 10.1038/s41467-022-33329-2 (PMC9508189; doi:10.1038/s41467-022-33329-2)
Supplement: Supplementary file 1 — Supplementary Information [file 41467_2022_33329_MOESM1_ESM.pdf]

Supporting Information for

Strain-regulated Gibbs Free Energy Enables Reversible Redox Chemistry of Chalcogenides for Sodium Ion Batteries

Minxia Jiang[1,3], Yingjie Hu[2,3], Baoguang Mao[1]*, Yixin Wang[1], Zhen Yang[1], Tao Meng[1], Xin Wang[1], Minhua Cao[1]*

[1]Key Laboratory of Cluster Science, Ministry of Education of China, Beijing Key Laboratory of Photoelectronic/Electrophotonic Conversion Materials, School of Chemistry and Chemical Engineering, Beijing Institute of Technology, Beijing 100081, P. R. China

[2]Nanjing Key Laboratory of Advanced Functional Materials, Nanjing Xiaozhuang University, Nanjing 211171, P. R. China

[3]These authors contributed equally: Minxia Jiang, Yingjie Hu.

E-mail: 7520200168@bit.edu.cn, caomh@bit.edu.cn

Supplementary Note 1. Kinetics calculations.

Based on the data of Warburg resistances, sodium ion diffusion coefficient (D_{Na}) can be calculated by the following equations[1]:

$$D_{Na}=\frac{R^2T^2}{2A^2n^4F^4C^2\sigma^2} \tag{1}$$

$$Z^{'}=R_D+R_L+\sigma\omega^{-1/2} \tag{2}$$

Here, R is the gas parameter, T is the absolute temperature, A is the surface area of the electrode material, n is the number of electrons transferred, F is the Faraday constant, C is the sodium ion concentration, and ω ($2\pi f$) is the low-frequency angular frequency. Additionally, σ is the Warburg factor related to charge diffusion, whose value can be obtained by fitting the real part Z' of the electrochemical impedance spectroscopy with $\omega^{-1/2}$.

The diffusion kinetics of Na^+ was further studied based on the CV measurement values. According to the Randles-Sevchik formula equation[2]:

$$I_p=2.69\times10^5An^{3/2}C_0D_{Na}{}^{1/2}v^{1/2} \tag{1}$$

Among them, I_p represents the peak current value, n represents the number of electrons transferred in each redox reaction, A is the surface area of the anode material, C_0 represents the lithium ion molar concentration in the anode material, and v is the scan rate of the CV curve. It can be seen from the formula that the redox peak current (I_p) has a linear relationship with the power of one-half of the sweep speed ($v^{1/2}$), and the slope of the straight line is proportional to D_{Na}. So the larger the slope, the greater the D_{Na} value, the faster the sodium ion diffusion kinetics during charge and discharge.

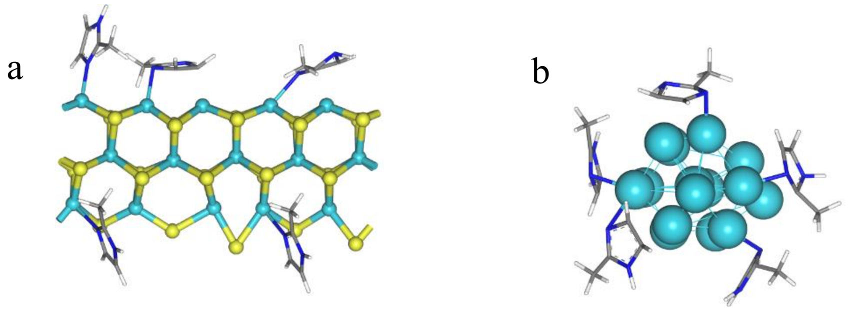

Supplementary Figure 1. The binding energies of 2-MI on $MoSe_2$ (a) and Mo (b).

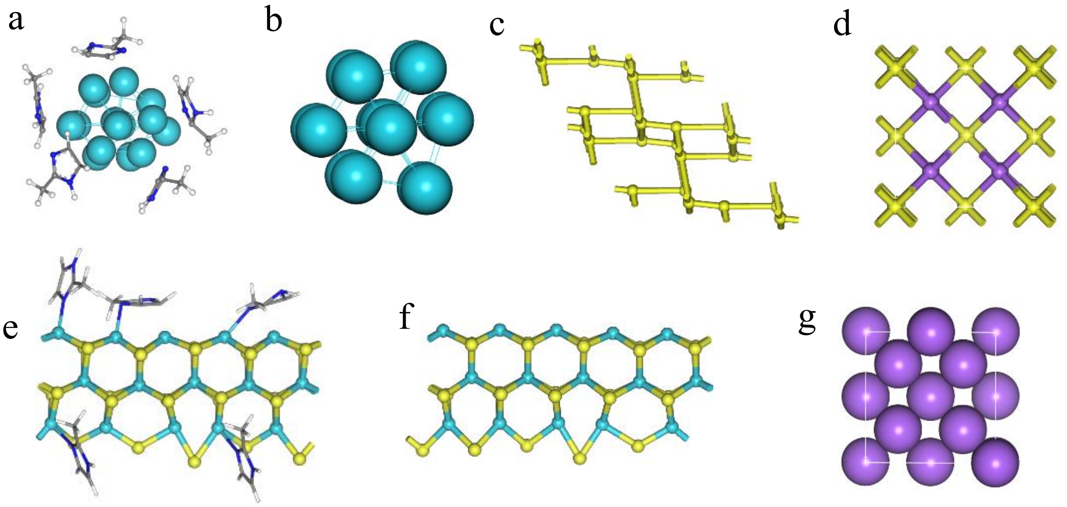

Supplementary Figure 2. The optimized structural models of (a) TS-Mo, (b) Mo, (c) Se, (d) Na_2Se, (e) TS-$MoSe_2$, (f) $MoSe_2$ and (g) Na for the calculations of ΔG.

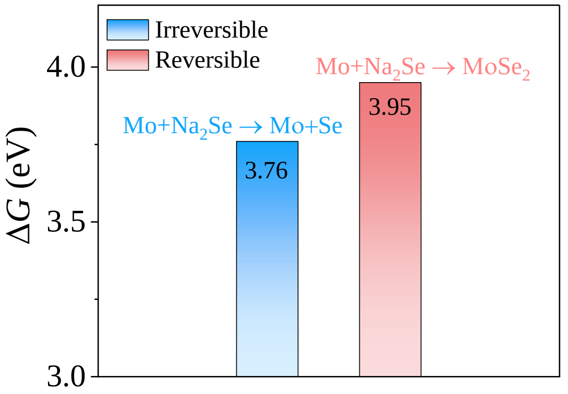

Supplementary Figure 3. The Δ*G* values per formula unit of two different reaction pathways for the discharged products of unstrained $MoSe_2$ (Mo and Na_2Se) during the charging process.

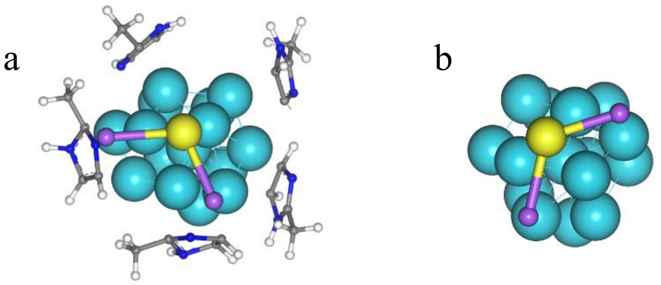

Supplementary Figure 4. Na_2Se adsorption on TS-Mo (a) and Mo (b).

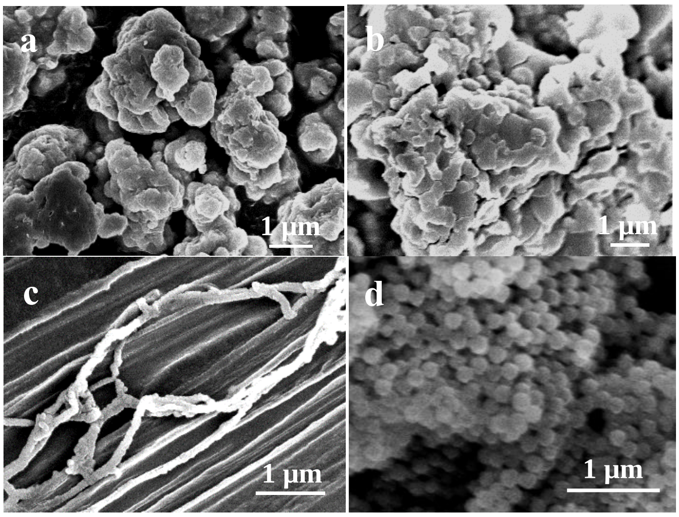

Supplementary Figure 5. FE-SEM images of the selenium powder (a), POM (b), 2-MI (c), and the synthesized Mo-precursor (d).

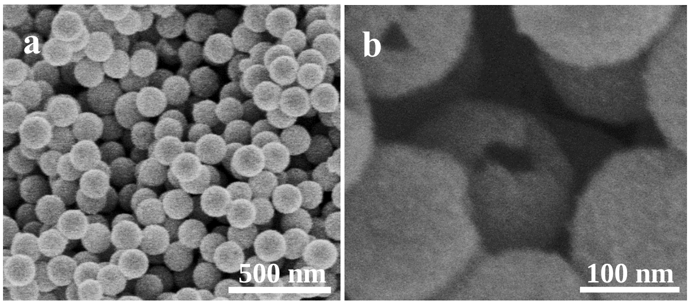

Supplementary Figure 6. (a,b) FE-SEM images of TS-$MoSe_2$ with different magnifications.

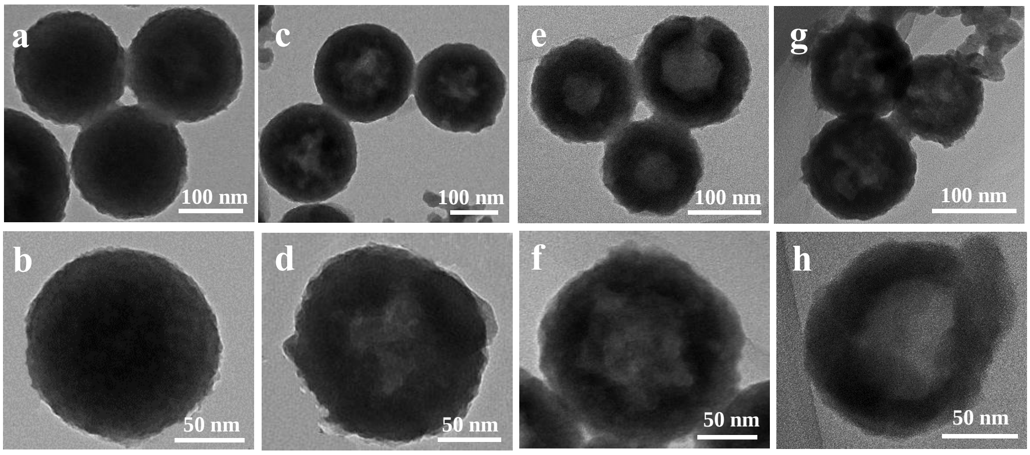

Supplementary Figure 7. TEM images of TS-$MoSe_2$ prepared with different reaction durations: (a,b) 2 h, (c,d) 6 h, (e,f) 12 h, and (g,h) 18 h.

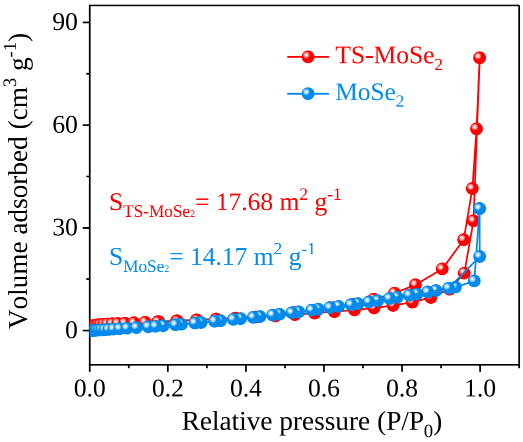

Supplementary Figure 8. The nitrogen adsorption-desorption isotherm curves of TS-$MoSe_2$ and $MoSe_2$.

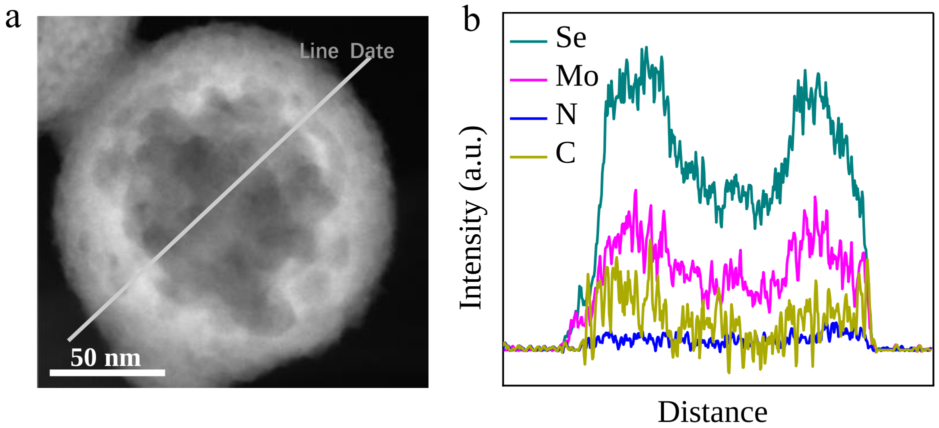

Supplementary Figure 9. (a,b) HAADF-STEM image of TS-$MoSe_2$ and corresponding line-scan profiles.

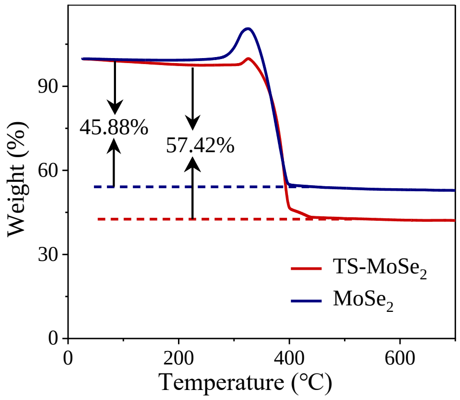

Supplementary Figure 10. TG curves of TS-$MoSe_2$ and $MoSe_2$.

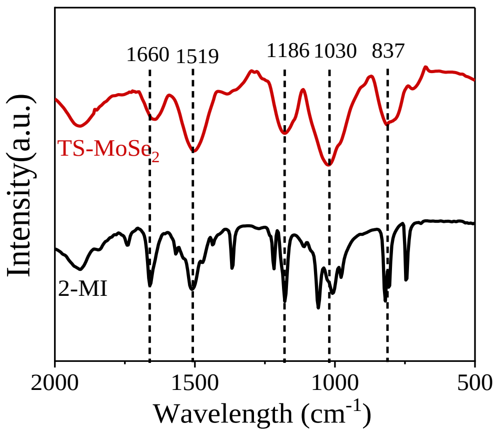

Supplementary Figure 11. FT-IR spectra of TS-$MoSe_2$ and 2-MI.

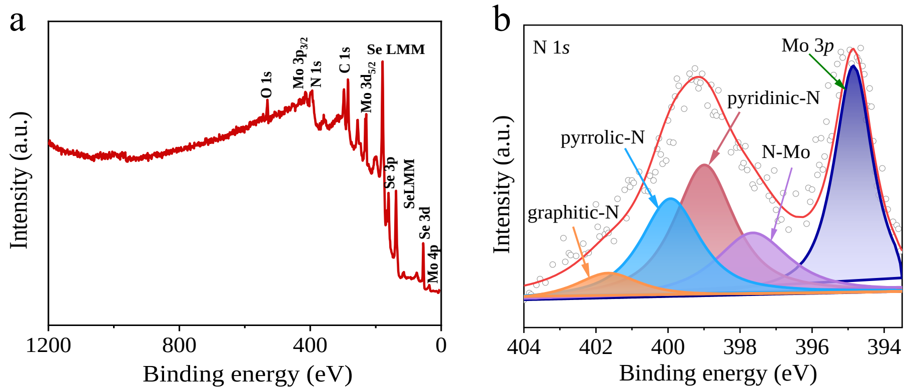

Supplementary Figure 12. Survey XPS spectrum (a) and high-resolution N 1*s* spectrum of TS-$MoSe_2$ (b).

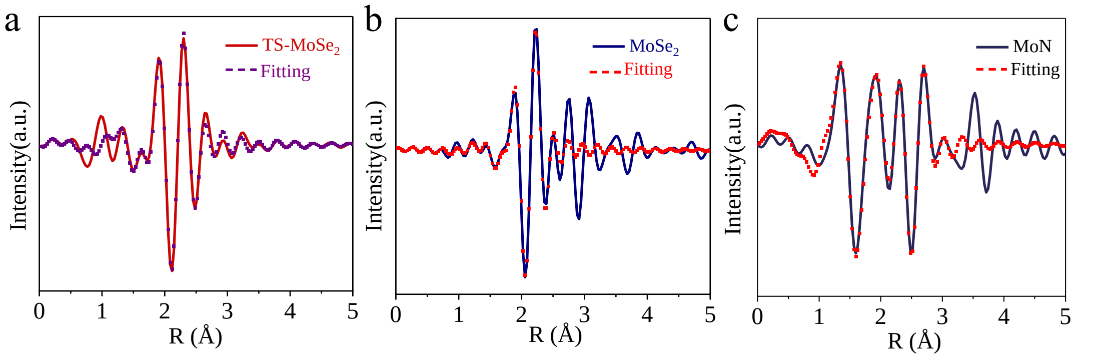

Supplementary Figure 13. k^2-weighed EXAFS function and corresponding fitting curves for TS-$MoSe_2$ (a), $MoSe_2$ (b) and MoN (c).

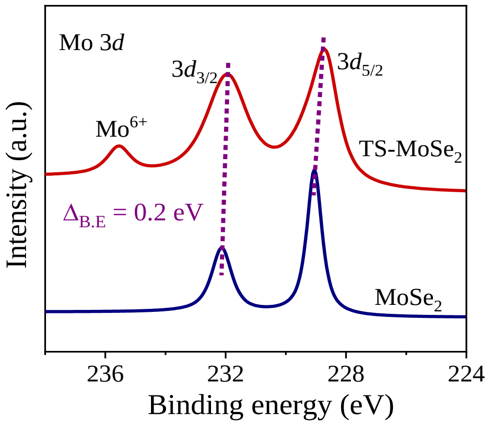

Supplementary Figure 14. High-resolution Mo 3*d* XPS spectra of TS-$MoSe_2$ and $MoSe_2$. The presence of Mo^{6+} in TS-$MoSe_2$ is caused by the surface oxidation during the testing process.

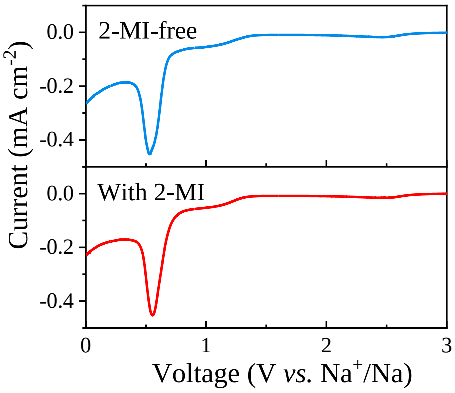

Supplementary Figure 15. LSV curves of the electrolyte with 2-MI and 2-MI-free.

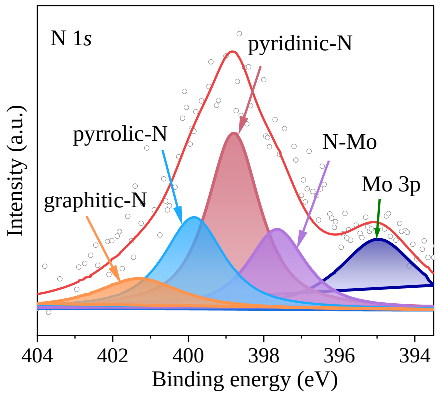

Supplementary Figure 16. High-resolution N 1*s* XPS spectra of the fully discharged (D0.01) product of TS-$MoSe_2$.

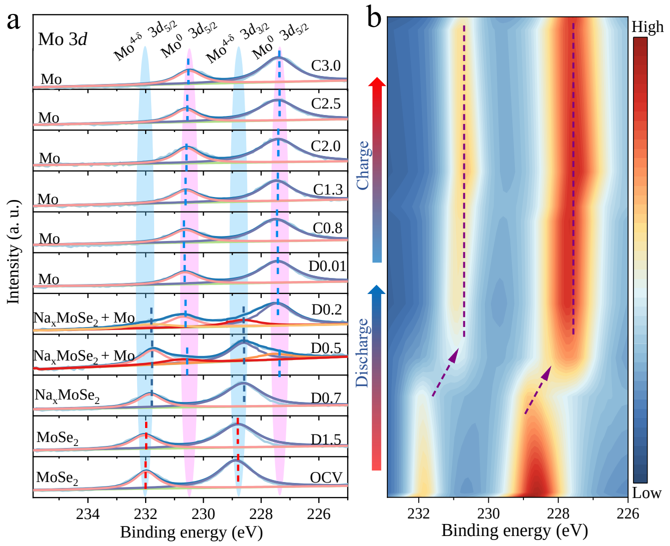

Supplementary Figure 17. Ex situ Mo 3*d* XPS spectra of $MoSe_2$ during the initial discharging and charging processes (a) as well as the corresponding mapping image (b).

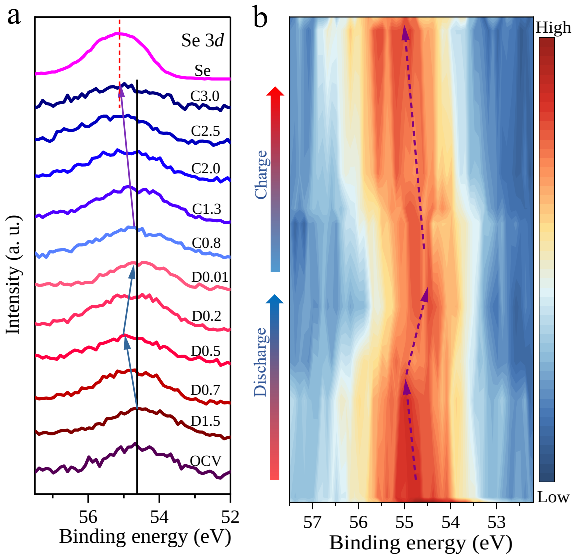

Supplementary Figure 18. Ex situ Se 3*d* XPS spectra (a) and corresponding mapping image (b) of $MoSe_2$ during the initial discharging and charging processes.

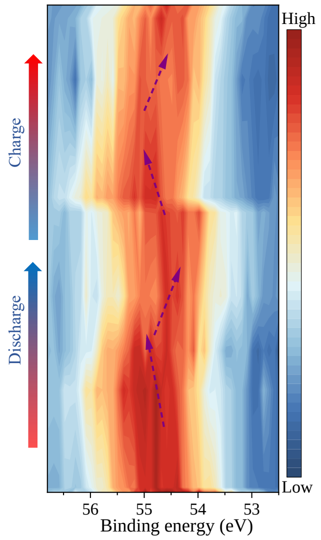

Supplementary Figure 19. Ex situ Se 3*d* XPS mapping image of TS-$MoSe_2$ during the initial discharging and charging processes.

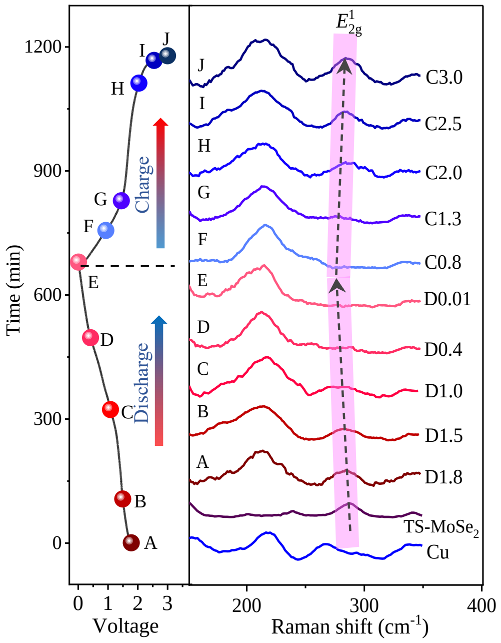

Supplementary Figure 20. In situ Raman spectra of TS-$MoSe_2$ during the initial discharging and charging processes (the experimental results from the repeated test of in situ Raman).

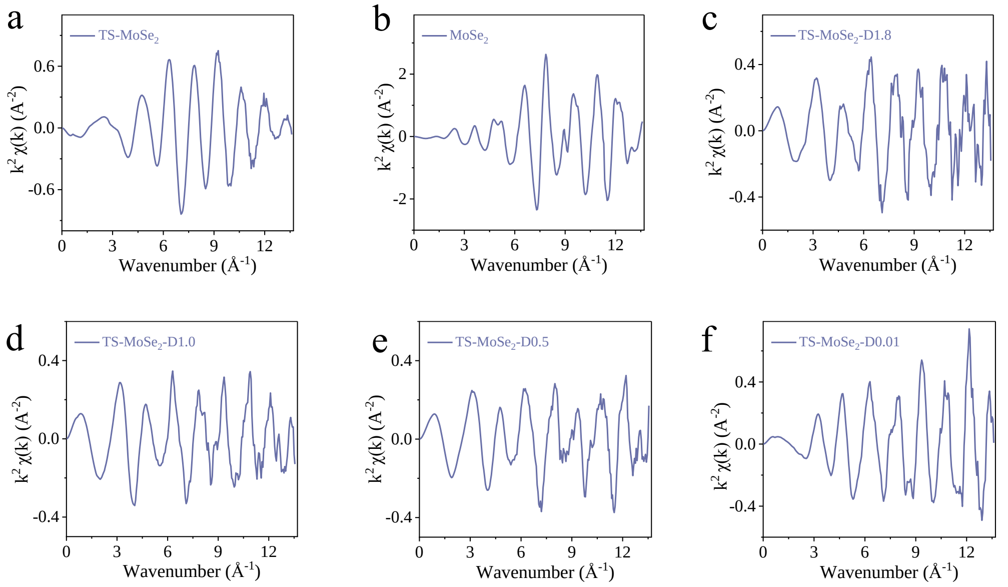

Supplementary Figure 21. The k-space XANES spectra of TS-$MoSe_2$ (a), $MoSe_2$ (b), and TS-$MoSe_2$ at different discharged states (c-f) for Mo K-edge.

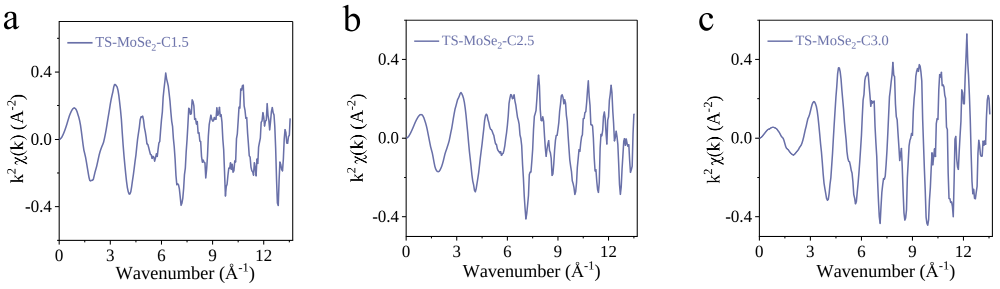

Supplementary Figure 22. The k-space XANES spectra of TS-$MoSe_2$ at different charged states (a-c) for Mo K-edge.

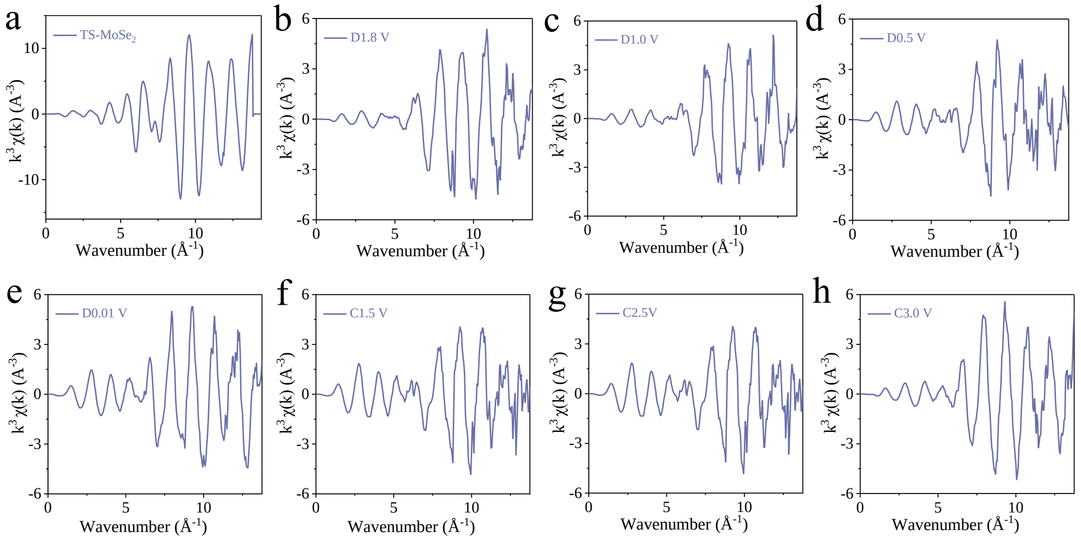

Supplementary Figure 23. The k-space XANES spectra of TS-$MoSe_2$ (a) and TS-$MoSe_2$ during the first discharge (b-e) state and charge (f-h) state for Se K-edge.

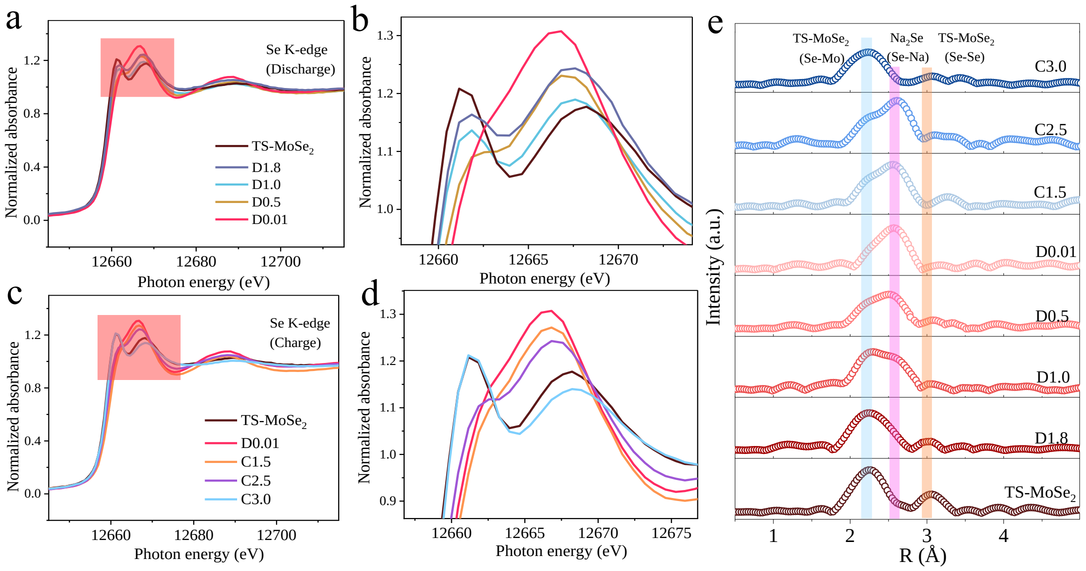

Supplementary Figure 24. Ex situ Se K-edge XANES spectra of TS-$MoSe_2$ during the first (a,b) discharging and charging states (c,d). (e) The corresponding EXAFS spectra.

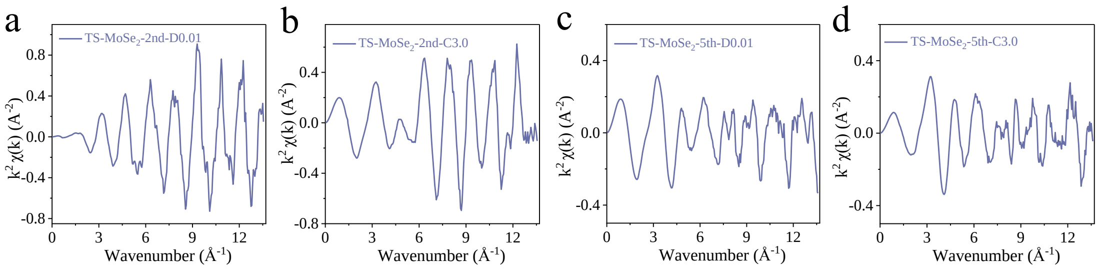

Supplementary Figure 25. The k-space XANES spectra of TS-$MoSe_2$ after the second (a,b) and fifth (c,d) cycles for Mo K-edge.

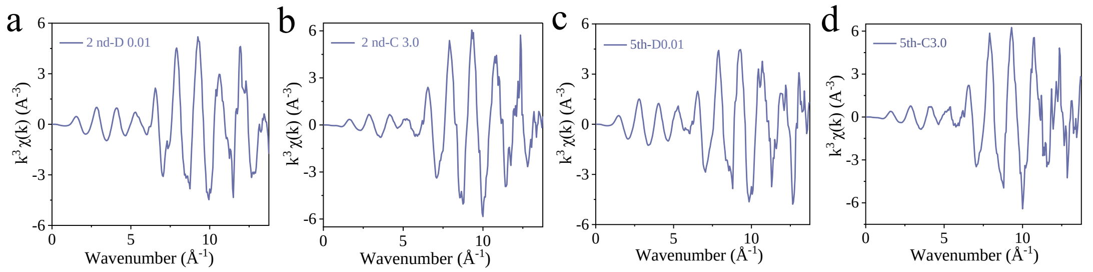

Supplementary Figure 26. The k-space XANES spectra of TS-$MoSe_2$ after the second (a,b) and fifth (c,d) cycles for Se K-edge.

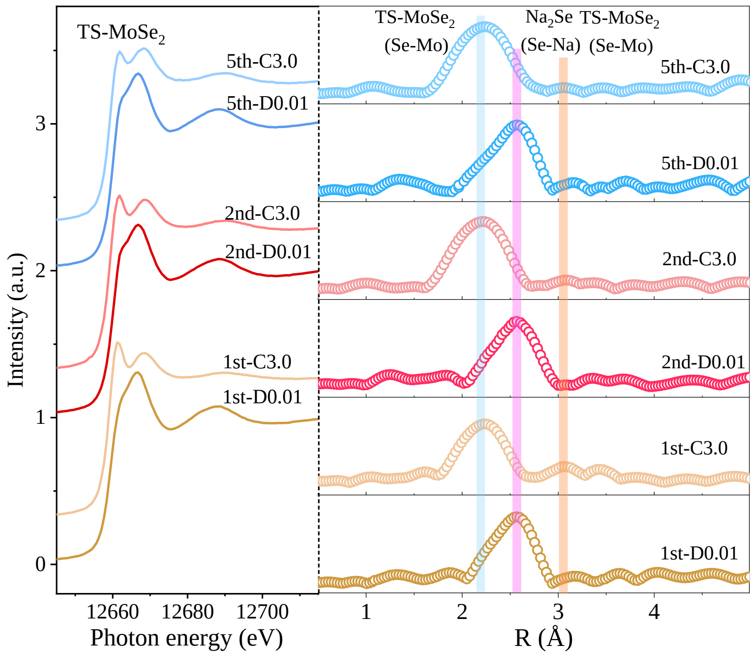

Supplementary Figure 27. Se K-edge XANES and EXAFS spectra of TS-$MoSe_2$ after the first, second, and fifth cycles.

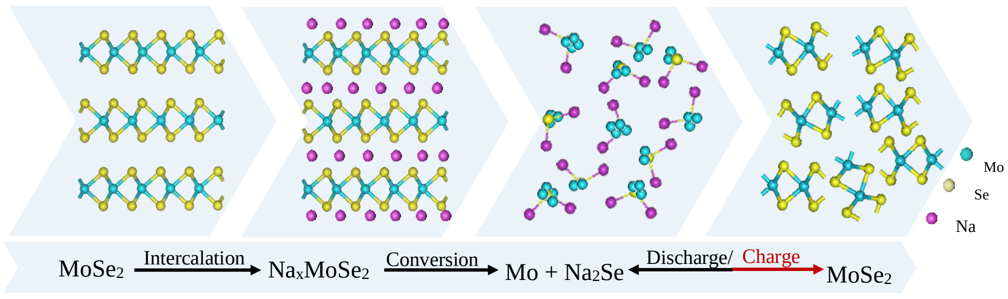

Supplementary Figure 28. Illustration of the sodium storage process for TS-$MoSe_2$.

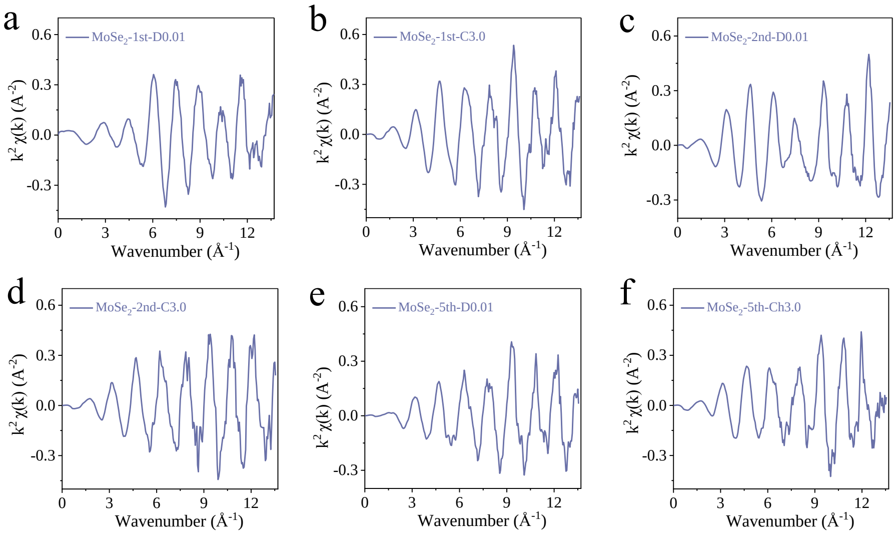

Supplementary Figure 29. The k-space XANES spectra of $MoSe_2$ after the first (a,b), second (c,d), and fifth (e,f) cycles for Mo K-edge.

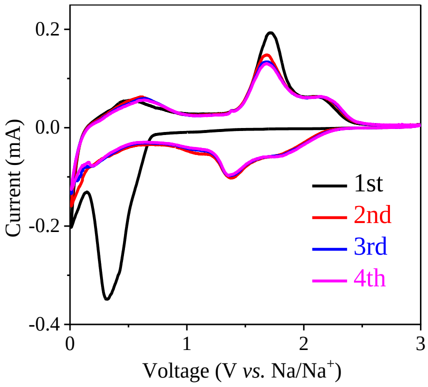

Supplementary Figure 30. CV curves of $MoSe_2$ between 0.01 and 3.0 V at a potential sweep speed of 0.1 mV s^{-1}.

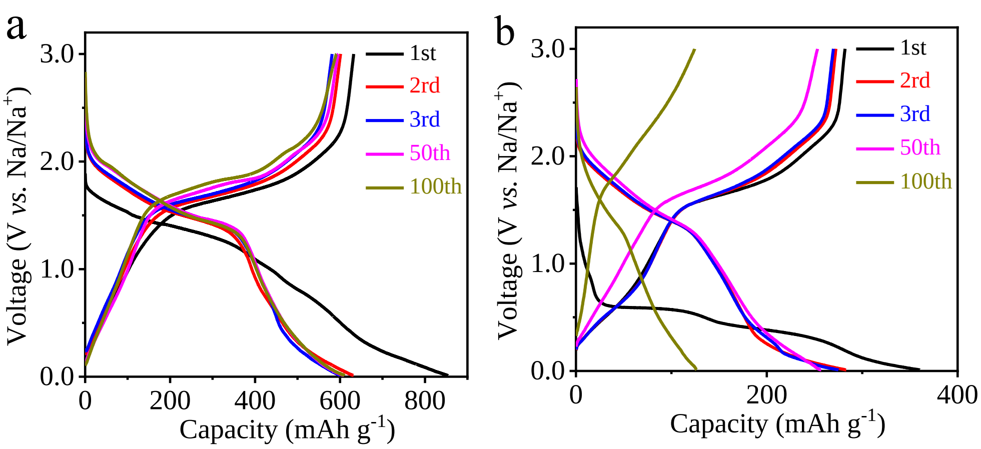

Supplementary Figure 31. Galvanostatic charge-discharge curves of (a) TS-$MoSe_2$ and (b) $MoSe_2$ at the current density of 0.1 A g^{-1} for the 1st, 2nd, 3rd, 50th, and 100th cycles.

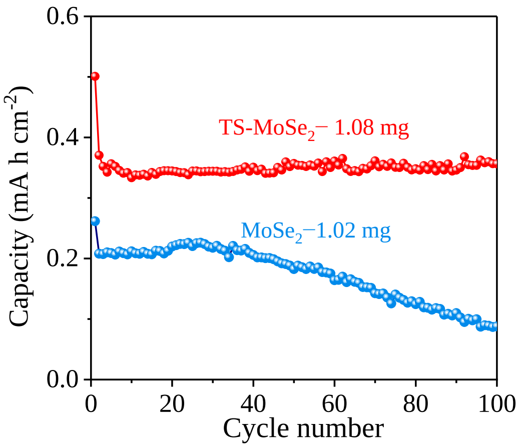

Supplementary Figure 32. The areal capacities of TS-$MoSe_2$ and $MoSe_2$ electrodes at the current density of 0.1 A g^{-1}.

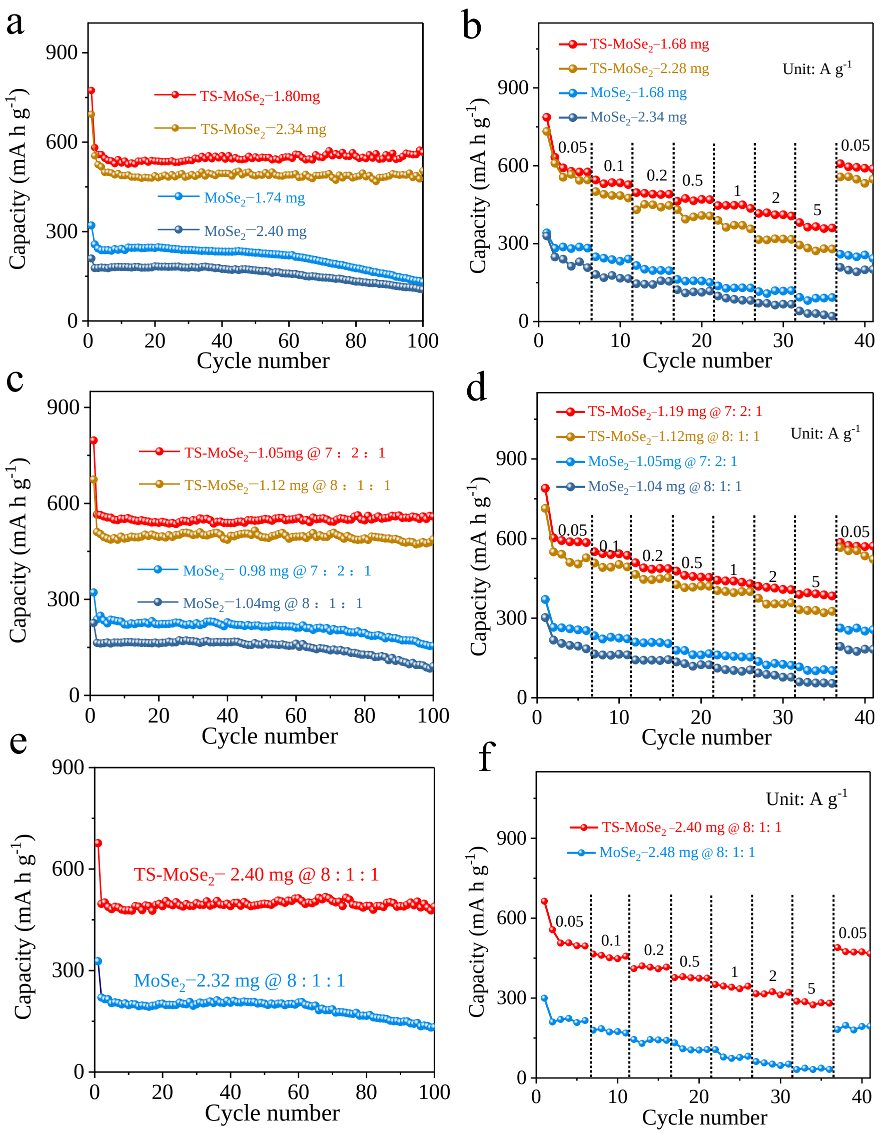

Supplementary Figure 33. Cycling and rate performances of TS-$MoSe_2$ and $MoSe_2$ with increased loadings of the active materials (a,b), different mass ratios of active materials, carbon black and binder (c,d), as well as both increased loading and decreased carbon black content (e,f), respectively.

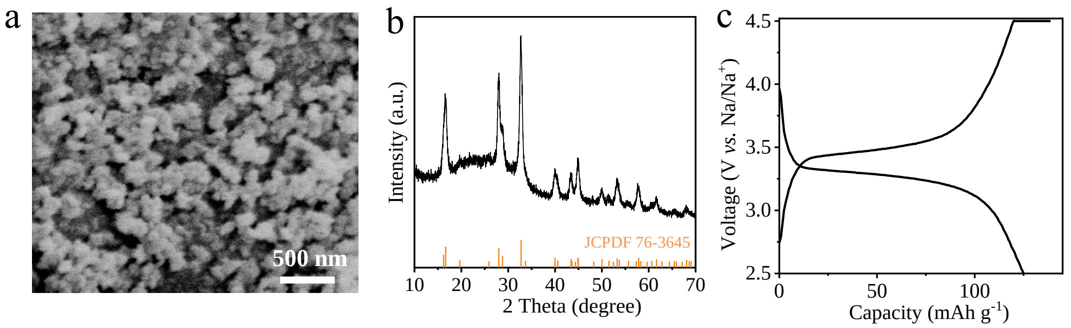

Supplementary Figure 34. (a) FE-SEM image, (b)XRD pattern and (c) galvanostatic charge-discharge curve of NVPOF.

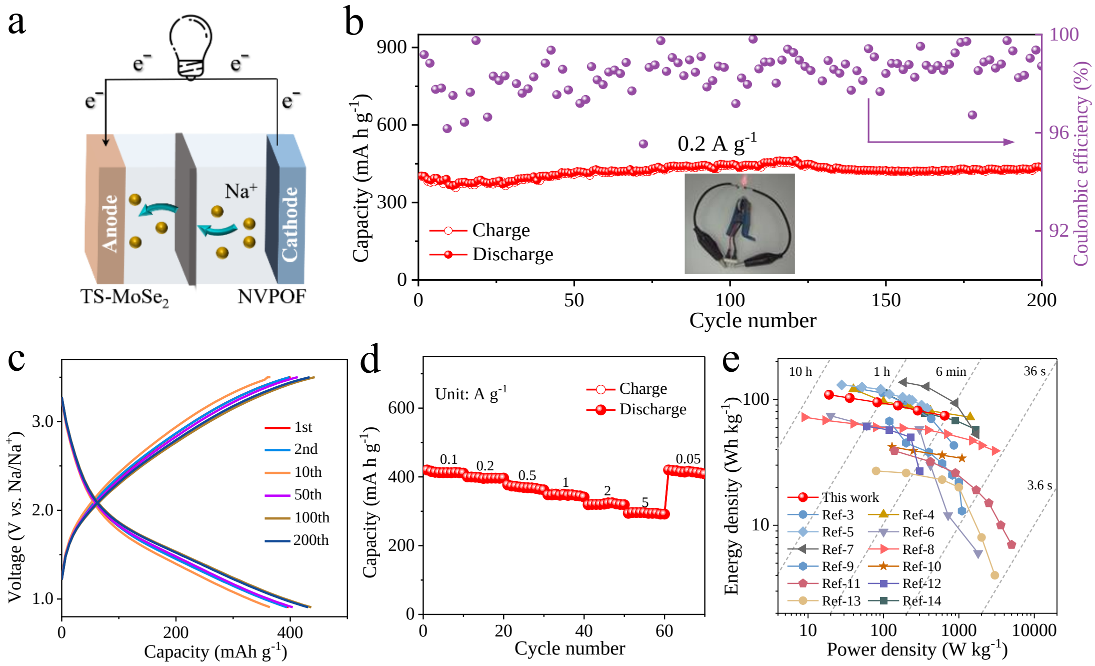

Supplementary Figure 35. Electrochemical performances of the TS-$MoSe_2$//NVPOF full cell. (a) Schematic illustration of the TS-$MoSe_2$//NVPOF full cell. (b) Cycling performance at 0.2 A g^{-1} and (c) corresponding galvanostatic charge-discharge curves. (d) Rate performance. (e) Ragone plots of gravimetric energy density *vs.* power density (**Ref-3:** SnP_2O_7/rGO//$Na_3V_2(PO_4)_3$/C; **Ref-4:** $MoSe_2$/C//NVP/C; **Ref-5:** graphite//prussian white; **Ref-6:** $Na_2Fe_2(SO_4)_3$//Ti_2CT_x; **Ref-7:** $CoSe_2$@NC//NVPOF; **Ref-8:** NTP@rGO//$Na_3V_2(PO_4)_3$/C; **Ref-9:** CNT//$Na_3V_2(PO_4)_3$, **Ref-10**: $NaTi_2(PO_4)_3$//Na_2PB; **Ref-11:** Na-ion V_2O_5-CNT//activated carbon; **Ref-12:** graphite//$Na_{0.7}CoO_2$; **Ref-13:** $NaTi_2(PO_4)_3$//$Na_{0.44}MnO_2$; **Ref-14:** $MoSe_2$/N,P-rGO//$Na_3V_2(PO_4)_3$ /C).

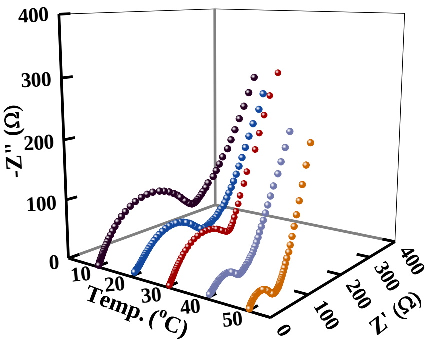

Supplementary Figure 36. Temperature-dependent Nyquist plots of $MoSe_2$.

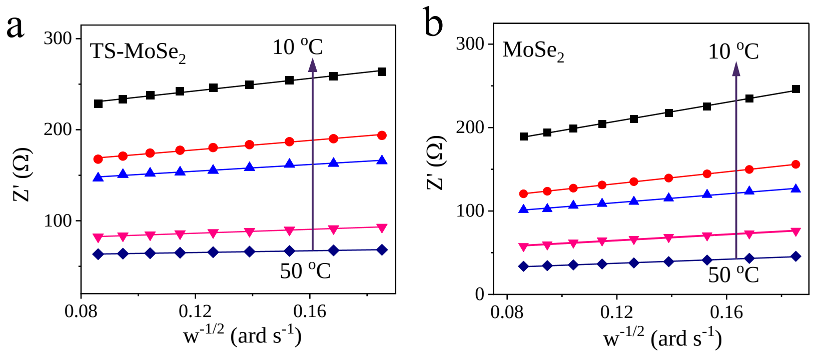

Supplementary Figure 37. The linear relationship of Z' *vs.* $\omega^{-1/2}$ for TS-$MoSe_2$ (a) and $MoSe_2$ (b).

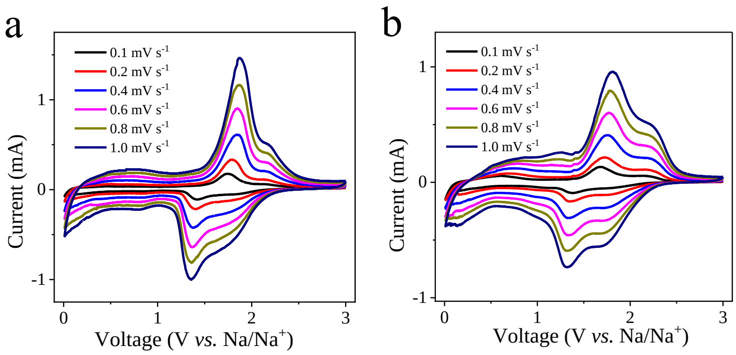

Supplementary Figure 38. CV curves of TS-$MoSe_2$ (a) and $MoSe_2$ (b) between 0.01 and 3.0 V at various scan rates from 0.1 to 1 mV s^{-1}.

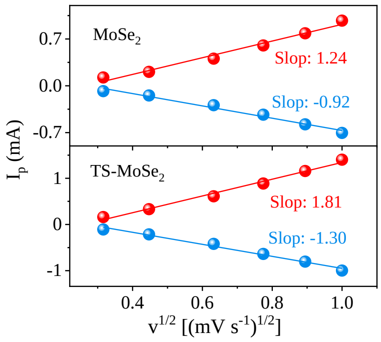

Supplementary Figure 39. Relationship between the peak current (I_p) and the square root of scan rate ($v^{1/2}$) of $MoSe_2$ and TS-$MoSe_2$.

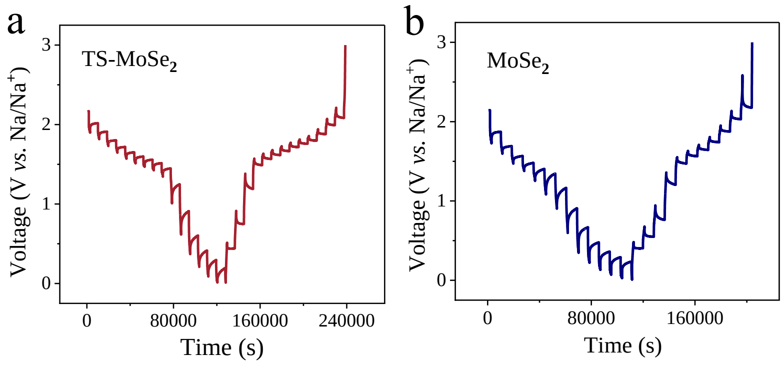

Supplementary Figure 40. GITT curves of TS-$MoSe_2$ (a) and $MoSe_2$ (b).

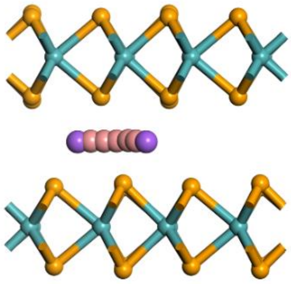

Supplementary Figure 41. The side view of transmission pathway of Na^+.

Table S1. The detailed calculation data of free energies during the reaction.

Reaction	ΔG (eV)	ΔE (eV)	ZPE (eV)	TΔS (eV)
TS-Mo + $2Na_2Se \rightarrow$ TS-$MoSe_2$ + 4Na + $4e^-$	3.0608	3.0757	0.0417	0.0566
Mo + $Na_2Se \rightarrow MoSe_2$ + 4Na + $4e^-$	3.9507	3.9302	0.0517	0.0312
Mo + $Na_2Se \rightarrow$ Mo + Se + 2Na + $2e^-$	3.7609	3.7929	-0.0016	0.0304

Table S2. CHN analysis results of TS-$MoSe_2$.

Samples \ Element	C (wt.%)	H (wt.%)	N (wt.%)
TS-$MoSe_2$	2.22	1.51	1.40

According to the chemical formula of the 2-MI, $C_4H_6N_2$, the content of the 2-MI is calculated by the following equation,

$$\omega_{2\text{-MI}}\% = \frac{\omega_{\text{element N}} \times M_{2\text{-MI}}}{M_{\text{element N}} \times 2} \times 100$$

Where the $\omega_{2\text{-MI}}$ and $\omega_{\text{element N}}$ are the mass fraction of the 2-MI and the element nitrogen, respectively. $M_{2\text{-MI}}$ and $M_{\text{element N}}$ are the molar mass of the 2-MI (82 g/mol) and the element nitrogen (14 g/mol), respectively. And thus, the mass loading of 2-MI could be determined to be 4.10 wt.% ($\frac{1.40\%}{14 \times 2} \times 82 \times 100 = 4.10\%$).

Table S3. Structural parameters of the sample obtained from the XAFS fitting.

Samples	Path	N	R (Å)	ΔE_0 (eV)	σ^2 [$Å^2$]	R-factor (%)
$MoSe_2$	Mo-Se	6.0	2.56±0.02	0.40	0.0050±0.00026	0.71
TS-$MoSe_2$	Mo-N	0.8	1.98±0.04	0.80	0.0053±0.00077	2.15
	Mo-Se	4.2	2.52±0.01	0.80	0.0058±0.00019	
MoN	Mo-N1	3.0	2.04±0.09	1.98	0.0064±0.00033	1.89
	Mo-N2	3.0	2.10±0.08	1.98	0.0051±0.00033	
	Mo-Mo1	2.0	2.90±0.07	1.98	0.0059±0.00012	
	Mo-Mo2	6.0	2.96±0.09	1.98	0.0058±0.00024	

N: coordination numbers; R: bond distance; σ^2: Debye-Waller factors; ΔE_0: the edge-energy shift; R factor: goodness of the fitting; S_o^2 is the amplitude reduction factor. S_0^2 for TS-$MoSe_2$ is 0.81, as obtained by fitting bulk $MoSe_2$. The coordination number error is about ±20%.

Table S4. R_{ct} based on the equivalent circuit fitting of impedance spectrum.

Samples \ Temperature (°C)	50	40	30	20	10
TS-$MoSe_2$	35.55	49.48	82.52	139.9	205
$MoSe_2$	53.85	98.67	177.9	308.4	600.1

Supplementary References

1. Wang X., et al. Surface charge engineering for covalently assembling three-dimensional MXene network for all-climate sodium ion batteries. *ACS Appl. Mater. Inter.* **12**, 39181−39194 (2020).
2. Wang J., et al. Mechanochemistry-induced biaxial compressive strain engineering in MXenes for boosting lithium storage kinetics. *Nano Energy* **87**, 106053 (2021).
3. Pan J., et al. SnP_2O_7 covered carbon nanosheets as a long-life and high-rate anode material for sodium-ion batteries. *Adv. Funct. Mater*. **28**, 1804672 (2018).
4. Liu Y., et al. Hierarchical nanoarchitectured hybrid electrodes based on ultrathin $MoSe_2$ nanosheets on 3D ordered macroporous carbon frameworks for high-performance sodium-ion batteries. *J. Mater. Chem. A* **8**, 2843-2850 (2020).
5. Patnaik S., Escher I., Ferrero G.A. & Adelhelm P. Electrochemical study of prussian white cathodes with glymes–pathway to graphite-based sodium-ion battery full cells. *Batteries & Supercaps* **5**, e202200043 (2022).
6. Wang X., et al. Pseudocapacitance of MXene nanosheets for high-power sodium-ion hybrid capacitors. *Nat. Commun.* **6**, 6544 (2015).
7. Geng J., et al. Topological transformation construction of a $CoSe_2$/N-doped carbon heterojunction with a three-dimensional porous structure for high-performance sodium-ion half/full batteries. *Inorg. Chem. Front.* **9**, 3176−3186 (2022).
8. Fang Y., et al. 3D graphene decorated $NaTi_2(PO_4)_3$ microspheres as a superior high-rate and ultracycle-stable anode material for sodium ion batteries. *Adv. Energy Mater.* **6**, 1502197 (2016).
9. Li S., et al. Effect of carbon matrix dimensions on the electrochemical properties of $Na_3V_2(PO_4)_3$ nanograins for high-performance symmetric sodium-ion batteries. *Adv. Mater.* **26**, 3545−3553 (2014).
10. Wu X., Cao Y., Ai X., Qian J. & Yang H. A low-cost and environmentally benign aqueous rechargeable sodium-ion battery based on $NaTi_2(PO_4)_3$-$Na_2NiFe(CN)_6$ intercalation chemistry. *Electrochem. Commun.* **31**, 145−148 (2013).
11. Zheng C., et al. High-performance sodium-ion pseudocapacitors based on hierarchically porous nanowire composites. *ACS Nano* **6**, 4319–4327 (2012).
12. Hasa I., et al. A sodium-ion battery exploiting layered oxide cathode, graphite anode and glyme-based electrolyte. *J. Power Sources* **310**, 26−31 (2016).

13. Li Z., Young D., Xiang K., Carter W. & Chiang Y.M. Towards high power high energy aqueous sodium-ion batteries: the $NaTi_2(PO_4)_3/Na_{0.44}MnO_2$ system. *Adv. Energy Mater.* **3**, 290−294 (2013).

14. Niu F, et al. $MoSe_2$-Covered N,P-Doped carbon nanosheets as a long-life and high-rate anode material for sodium-ion batteries. *Adv. Funct. Mater.* **27**, 1700522 (2017).
